# How do Health Care Providers Want to Learn? Shaping Brain Health and Dementia Professional Education

**DOI:** 10.1093/geroni/igaf122.4248

**Published:** 2025-12-31

**Authors:** Jennifer Pettis, Nancy Keach, Brooks Kenny, Lisa McGuire

**Affiliations:** Gerontological Society of America, Washington, District of Columbia, United States; BrightFocus Foundation, Clarksburg, Maryland, United States; BrightFocus Foundation, BrightFocus Foundation, Maryland, United States; Gerontological Society of America, Atlanta, Georgia, United States

## Abstract

The Gerontological Society of America (GSA) KAER Toolkit for Brain Health serves as a timely and impactful resource for educating health care professionals across disciplines about brain health and dementia. BrightFocus Foundation’s (BFF) Zoom in on Dementia & Alzheimer’s is a monthly information program that provides expert insights on key topics within the Alzheimer’s landscape. Despite the abundance of education content developed for healthcare professionals, little is known about professionals’ needs and preferences about clinical education. To ensure that educational content meets the needs of diverse healthcare professionals, GSA and BFF convened three virtual focus groups during July and August 2025. Thirty-three GSA members recruited from GSA’s Health Sciences Section participated. Focus groups included diverse interdisciplinary providers (e.g., doctors, nurses, social workers, physician associates, pharmacists, physical therapists). One-hour groups were conducted by a trained facilitator, and each participant was provided with a small incentive for their time. Each group was queried about their needs and preferences regarding delivery, length, and format for clinical education and content distribution channels or sources where providers obtain their clinical education. Participants were also asked to provide input about topics related to dementia. The focus groups yielded valuable insights about members’ needs and preferences around educational programming for dementia including priority areas aligned in each step in the GSA KAER—Kickstart, Assess, Evaluate, Refer—framework and the Zoom In educational series. This presentation will highlight findings from the focus groups of healthcare professionals and describe educational strategies resulting from the findings.

